# Supplementary material for: The Relative Roles of Ambient Temperature and Mobility Patterns in Shaping the Transmission Heterogeneity of SARS-CoV-2 in Japan
Source: Viruses. 2022 Oct 11;14(10):2232. doi: 10.3390/v14102232 (PMC9611010; doi:10.3390/v14102232)
Supplement: Supplementary file 1 [file viruses-14-02232-s001.zip › viruses-1944601-supplementary.pdf]

viruses

MDPI

Supplementary Files for: The Relative Roles of Ambient Temperature and Mobility Patterns in Shaping the Transmission Heterogeneity of SARS-CoV-2 in Japan

Keita Wagatsuma [1,2,*], Iain S. Koolhof [1,3] and Reiko Saito [1]

[1] Division of International Health (Public Health), Graduate School of Medical and Dental Sciences, Niigata University, Niigata 951-8510, Japan
[2] Japan Society for the Promotion of Science, Tokyo 102-0083, Japan
[3] College of Health and Medicine, School of Medicine, University of Tasmania, Hobart 7000, Australia
* Correspondence: waga@med.niigata-u.ac.jp; Tel.: +81-25-227-2129

1. Materials and Methods

1.1. Mathematical Framework of Estimating Time-Dependent Transmissibility

In the present study, we estimated the time-dependent effective reproductive number R_t (defined as the expected number of secondary cases arising from a single primary case at calendar time *t*) following the Bayesian framework applied to the simple branching process models proposed by Cori et al. [1]. The model is an extension of Fraser's method [2] and is widely adopted in infectious disease epidemiological study [3-5]. Fraser proposed a time-varying estimate of temporal R_t from the deterministic renewal equation of an epidemic as:

$$R_t = \frac{I_t}{\sum_{k=0}^{m} w_k I_{t-k}} \quad (1.1)$$

where I_t is the number of incident symptomatic cases (i.e., notifications) between time *t* and time *t* + 1, and w_k is the serial interval distribution such that $\sum_{k=0}^{m} w_k = 1$. The serial interval distribution was assumed to be constant during the epidemic. A Bayesian framework was developed to generalize this inferential approach and to account for the inherent stochasticity of the transmission process, assuming that the expected incidence at time t ($R_t \sum_{k=0}^{m} w_k I_{t-k}$), is a Poisson-distributed count. Consider that the transmissibility is constant over the time window $[t-\tau, t]$ and is denoted by $R_{[t-\tau,t]}$; then, the likelihood of $I_{t-\tau}, \dots\dots\dots, I_t$ given $R_{[t-\tau,t]}$ and $I_0, \dots\dots\dots, I_{t-\tau-1}$ is as follows:

$$P(I_{t-\tau}, \dots\dots\dots, I_t \middle| I_0, \dots\dots\dots, I_{t-\tau-1}, w, R_{[t-\tau,t]}) = \prod_{s=t-\tau}^{t} \frac{e^{-R_{[t-\tau,t]}\Lambda_s} (R_{[t-\tau,t]}\Lambda_s)^{I_s}}{I_s!} \quad (1.2)$$

where, $\Lambda_s = \sum_{s=0}^{m} w_s I_{t-s}$. Assuming that Gamma (a, b) is a conjugate prior distribution for $R_{[t-\tau,t]}$, the posterior joint probability distribution of $R_{[t-\tau,t]}$ can be derived as

$$R_{[t-\tau,t]}{}^{a+\sum_{s=t-\tau}^{t} I_s - 1} e^{-R_{[t-\tau,t]}\left(\sum_{s=t-\tau}^{t} \Lambda_s + \frac{1}{b}\right)} \prod_{s=t-\tau}^{t} \frac{\Lambda_s{}^{I_s}}{I_s!}. \quad (1.3)$$

Equation 1.3 indicates the posterior distribution of $R_{[t-\tau,t]}$ is a gamma probability distribution with parameters $(a + \sum_{s=t-\tau}^{t} I_s, (\sum_{s=t-\tau}^{t} \Lambda_s + \frac{1}{b})^{-1})$.

1.2. Serial Interval Probability Distribution and Smoothing Window

Theoretically, epidemiological information regarding the generation time, which is defined as the period between the infection of the index and the next case, is needed; however, this information is usually difficult to ascertain. Instead, the R_t value can be adjusted to include the serial interval, which is defined as the interval between the onset of the index and the next case, as an infectivity function, assuming a gamma distribution [1, 6]. In this present study, we used the serial interval (SI) (mean SI: 4.7 days, standard deviation SI: 2.9 days) described by Nishiura et al. [7]. The choice of smoothing window was determined by a trade-off between temporal resolution and credible interval width around the

resulting estimates, and our time-varying estimates were made with a seven-day sliding window.

1.3. Identification Strategy of the Time-Series Statistical Model

To establish the time-series statistical model, multiple stages were used to build a robust and reliable model. Prior to constructing the model, we checked the probability distribution of the dependent variable, the daily time-dependent R_t (the normality of probability distribution was assessed by the Shapiro-Wilk test) (Figure S2), and assessed the relationships (e.g., linearity) between daily time-dependent R_t and each independent variable. If found to be significantly non-normal to the dependent variable, the value was transformed, as appropriate to approximate symmetry, utilizing logarithmic scale transformation with a logarithmic-link probability function in the modelling process. Generally, assessing the transformation of dependent variables in time-series driven systems allows the variables to be assessed as stationary effects and often improves forecasting accuracy. All dependent and independent variables included in the statistical models were assessed for multicollinearity using the pairwise Spearman's rank-order cross-correlation coefficient (ρ). If the variables were found to be highly linearly correlated (cut-off of $|\rho| > 0.8$), the variable with the largest mean absolute statistical correlation with the other independent variables was removed [8]. In the preliminary analysis, no independent variables showed strong statistical linear correlations (Table S2).

In the present study, we conducted a retrospective time-series analysis of the transmission dynamics heterogenicity of the outbreak using classical standard time-series statistical modelling. Generally, the respiratory viruses transmissibility is influenced by the depletion of susceptible inter-epidemic effects as intrinsic drivers, along with the potential effects of extrinsic drivers (e.g., non-pharmaceutical interventions [NPIs] and meteorological drivers). Therefore, we formulated a standard time-series generalized additive model (GAMs) with a gamma probability distribution family and logarithmic-link probability function, allowing for overdispersion in the observational epidemiologic data, combined with distributed lag nonlinear models (DLNMs). This method was used to disentangle the underlying association between temporal transmissibility (i.e., time-varying R_t) and different potential behavioral and environmental drivers (i.e., mean ambient temperature and retail and recreation mobility) as the main exposures, as a primary model. Generally, GAMs are a semi-parametric extension of the generalized linear model (GLMs), which allows a more flexible description of exposure-response relationships between dependent variables that are attributable to the transmission dynamics of infectious diseases and environmental exposures. Notably, the results are assumed to depend on the sum of smoothed linear combinations of the dependent variable [9-13]. The class of DLNM models introduces a cross-basis function, which can be obtained by calculating the tensor product of the basis functions and describes the probability distribution of the dependent variable in the independent variable dimension and the lagging dimension to simultaneously assess the lag effect and non-linear effects of the time-varying exposure driver [14-16]. Indeed, these bidimensional exposure-lag-response associations represent the temporal change in risk after a specific exposure, and they estimate the probability distribution of immediate and delayed effects that accumulate across the lag period.

The cross-basis term for the DLNM acts as a basis function predictor in two dimensions: exposure space and lag space. For the exposure space, we chose the natural cubic spline function with three knots as the basis function, corresponding to the 25th, 50th, and 75th percentiles of the exposure distribution. To address for the possible harvesting and misalignment of effect delayed heterogeneity of behavioral and environmental drivers on the dynamics of SARS-CoV-2 transmission, based on previous accumulated literatures describing the incubation period of SARS-CoV-2 (approximately 1–14 days) [17-20] and potential reporting delay [21], we considered temporal lags (i.e., delays in potential effect) of up to 21 days as the default lag for cross-basis function of each independent variable (i.e., ambient mean temperature and retails and recreation mobility) related to the

dependent variable, accordingly. In the lag response dimension, we considered a natural spline with one internal knot as the basis function. These temporal time lags are useful for describing biologically and physically plausible time lag processes in the population dynamics and natural history of the SARS-CoV-2 host reservoir and the subsequent incubation period before disease notifications occur.

In this time-series statistical modeling study, we formulated a penalized smoothing thin plate spline function for relative humidity (continuous), precipitation (continuous), windspeed (continuous), and daily number of SARS-CoV-2 vaccinations (continuous) to control for nonlinear confounding effects of the meteorological variables and immune responses after vaccination (i.e., hybrid immunity). We also incorporated a prefectural-level fixed effect indicator variable during the state of emergency declaration period (category) to control the intensity of NPIs at the population level and to control for time-invariant prefectural characteristics in each prefecture or regional variation [22]. We used prefectural-level geographical (i.e., latitude and longitude in each prefecture) variables (continuous) to better control for spatiotemporal variations, fixed effect indicator variable of each SARS-CoV-2 variant in day t (i.e., four categories of the wild type, the Alpha [B.1.1.7] variant, Delta [B.1.617.2] variant, and Omicron [B.1.1.529] variant). The thin plate splines are data-driven, and the optimal degree of freedom (d.f.) (i.e., smoothing parameter) for the penalized smoothing plate spline function in the models was selected through minimization of the generalized cross validation (GCV) criterion. The best statistical model with a low GCV value and high prediction accuracy is preferred, as it achieves a more optimal combination of goodness-of-fit and parsimony. Additionally, we also modelled individual-varying baseline risk on top of shared long-term, seasonal variation and cycle, and weekly trends by incorporating natural cubic splines of time (seven degrees of freedom per year for the main analysis), day-of-week (category), and public holiday (category) fixed-effects variables as possible confounder [23-26]. Additionally, autocorrelation of residuals in the case of infectious disease is pathogen-specific and needs to be accounted for; therefore, autoregressive terms of order one (i.e., one day) were incorporated into the statistical models [27].

Formally, the general algebraic definition of time-series statistical models is formulated as follows, with further extensions attempted:

$$P(Y_t \mid R_t) \sim Gamma\left[exp\left(\alpha + \sum_{\tau} f_G(x_{\tau,t,i}) + \sum_{\mu} h_G(y_{\tau,t,i}) + S_{t,i} + P_i + La_i + Lo_i + \sum_i f(z_t^i;\theta) + D_t + H_t + V_t + min(w_{limit,i}, w_{t,i}) + O_t + ln(R_{t-1}) + \varepsilon_{t,i}\right)\right]$$

where Y_t is the outcome time series; R_t is the expected time series of the daily time-dependent effective reproductive number in prefecture i on day t; α corresponds to the overall intercept; and $f_G(x_{\tau,t,i})$ denotes the cross-basis function *with exposure and lag effects modelled by a natural cubic spline function and a linear function* of mean ambient temperature and retail and recreation mobility in prefecture i on day t, *respectively.* The notation $h_G(y_{\tau,t,i})$ represents a penalized smoothing spline function of relative humidity, precipitation, wind speed, and daily number of SARS-CoV-2 vaccinations, to control for nonlinear confounding effects (term of nonlinear). $S_{t,i}$ is a fixed effect indicator variable that takes the value of one during the state of emergency declaration period in prefecture i on day t and zero. Otherwise, P_i denotes prefectural characteristics or regional variable indicators in prefecture i. La_i and Lo_i denote latitude and longitude variables in prefecture i. $\sum_i f(z_t^i;\theta)$ denotes natural cubic splines of time (seven degrees of freedom per year). D_t denotes week-of-day at t. H_t denotes public holiday in day t, and V_t is a fixed effect indicator variable of each variant on day t. In this statistical model, $w_{t,i}$ is the degree of risk awareness in prefecture i on day t [28]. This was graded by assuming that it was linearly associated with the smoothed number of newly reported cases, following a positive association between confirmed cases and risk perception found in a study from the United Kingdom (UK), using longitudinal data [29]. The effect of this variable $w_{t,i}$ was capped at a predefined upper limit ($w_{limit,i}$) that corresponded to the government definition of the "highest alert

level" incidence in Japan (i.e., 25 confirmed cases per 100,000 population in a week) [30]. According to this definition, the daily number of cases giving an upper limit for each prefecture *i* ($w_{limit,i}$) was specified as 497 in Tokyo, 315 in Osaka, 270 in Aichi, 188 in Hokkaido, 182 in Fukuoka, and 52 in Okinawa. R_{t-1} denotes autoregressive terms of order one, accounting for potential serial correlation. O_t *denotes* the logarithm of the yearly population by prefecture as an offset term [31], and term $\varepsilon_{t,i}$ indicates the error term. To quantify the total contribution, independent effects, and relative importance of behavioral and environmental drivers, we included all variables in the same model. By including all variables of interest in the same regression equation, we strengthened the interpretation of the effects as independent and additive, based on accumulated empirical knowledge.

To test the sensitivity of the results to the modeling choices described above, we repeated the analysis by varying the d.f. of the natural cubic spline of time from seven d.f. per year to three or 11 d.f. per year. In addition, we assessed the sensitivity of redefining the lag-response dimension using a natural cubic spline and three equally placed internal knots. For all statistical models, we quantified estimates as cumulative days (i.e., 0–21 days) and single days (i.e., 0, 7, 14, and 21 days), and evaluated relative risks (RRs) together with their 95% confidence intervals (CIs) at the 1st, 25th, 75th, and 99th percentiles of the mean ambient temperature. Retails and recreation mobility were used to present the strengths of the associations. Because the associations were generally non-linear and delayed, we utilized the overall median value (50th percentile) of mean ambient temperature and the retail and recreation mobility as the reference levels. Furthermore, we described three-dimensional plots and exposure-response curves to observe the shape of the associations. Statistical significance was considered at a *p*-value of less than 0.05 (i.e., a type I error), on a two-tailed test. All analyses were performed using STATA version 15.1 statistical software (Stata Corp, College Station, TX, USA) and R statistical programming software version 4.1.0 (R Foundation for Statistical Computing, Vienna, Austria) using "dlnm" and "mgcv" [32, 33].

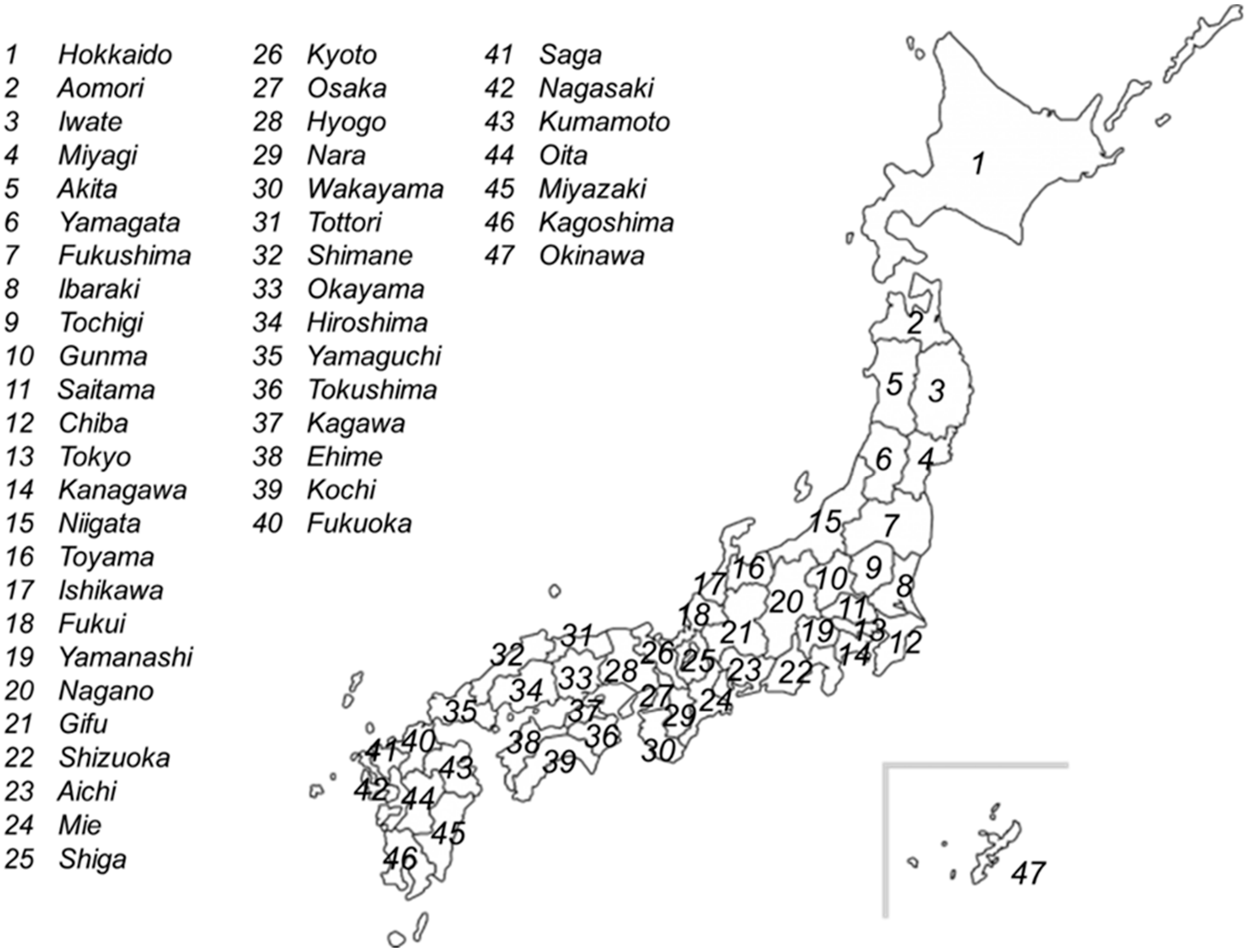

Figure S1. The geographic locations of the 47 Japanese prefectures. Japan is located at latitudes from approximately 26°N to 43°N and longitudes from approximately 127°E to 141°E, in the western pacific region, and constructed of a total of 47 Japanese prefectures (covering the whole country) from north to south: Hokkaido, Aomori, Iwate, Miyagi, Akita, Yamagata, Fukushima, Ibaraki, Tochigi, Gunma, Saitama, Chiba, Tokyo, Kanagawa, Niigata, Toyama, Ishikawa, Fukui, Yamanashi, Nagano, Gifu, Shizuoka, Aichi, Mie, Shiga, Kyoto, Osaka, Hyogo, Nara, Wakayama, Tottori, Shimane, Okayama, Hiroshima, Yamaguchi, Tokushima, Kagawa, Yamanashi, Nagano, Gifu, Shizuoka, Aichi, Mie, Shiga, Kyoto, Osaka, Hyogo, Nara, Wakayama, Tottori, Shimane, Okayama, Hiroshima, Yamaguchi, Tokushima, Kagawa, Ehime, Kochi, Fukuoka, Saga, Nagasaki, Kumamoto, Oita, Miyazaki, Kagoshima, and Okinawa.

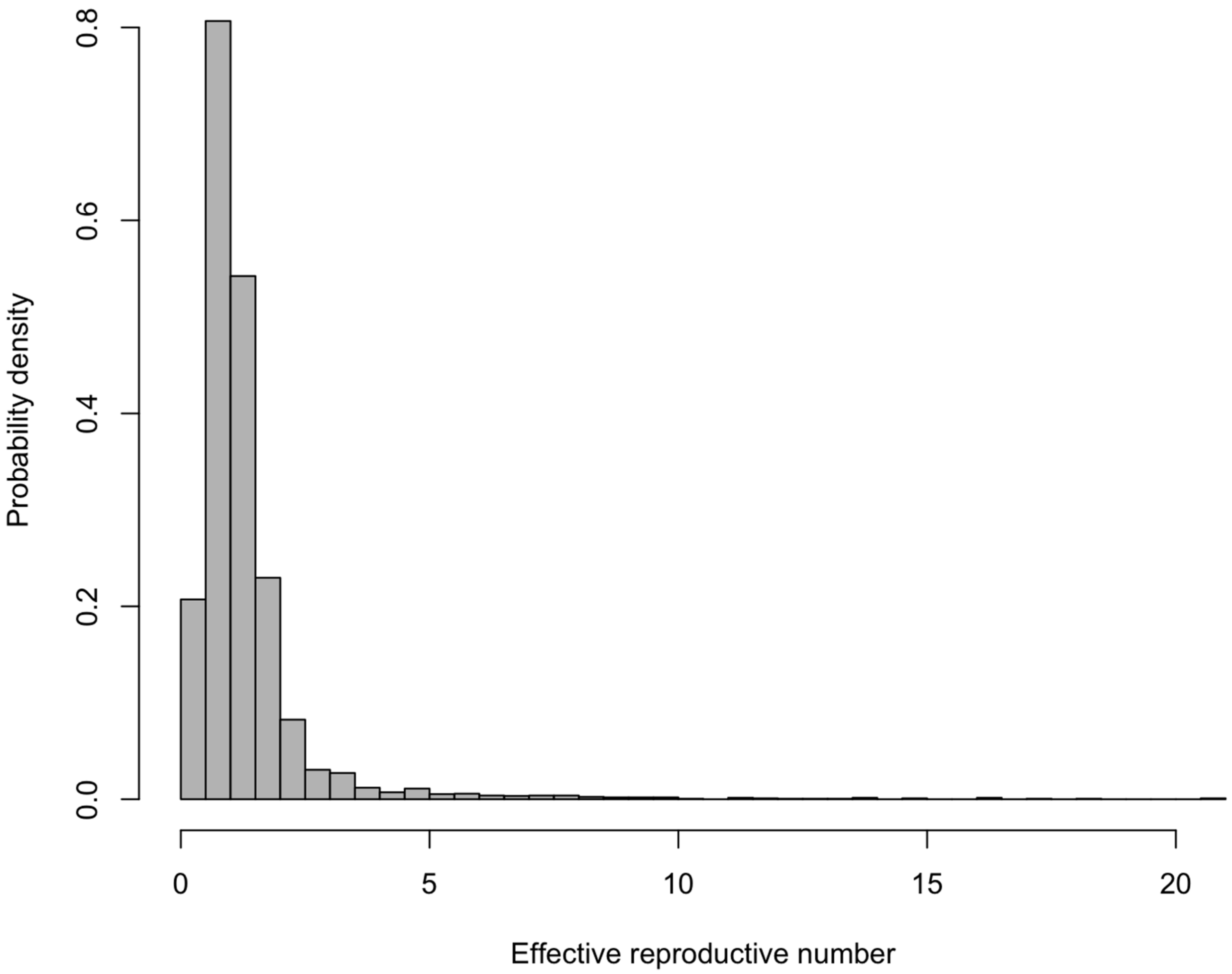

Figure S2. Probability distribution of the daily temporal effective reproductive numbers in Japan across all of the included prefectures throughout the study period. The mean daily temporal effective reproductive numbers in Japan across all the included prefectures and days was 1.30 (standard deviation [SD], 1.40). These observational data do not follow a normal distribution (Shapiro-Wilk test, $P < 0.001$).

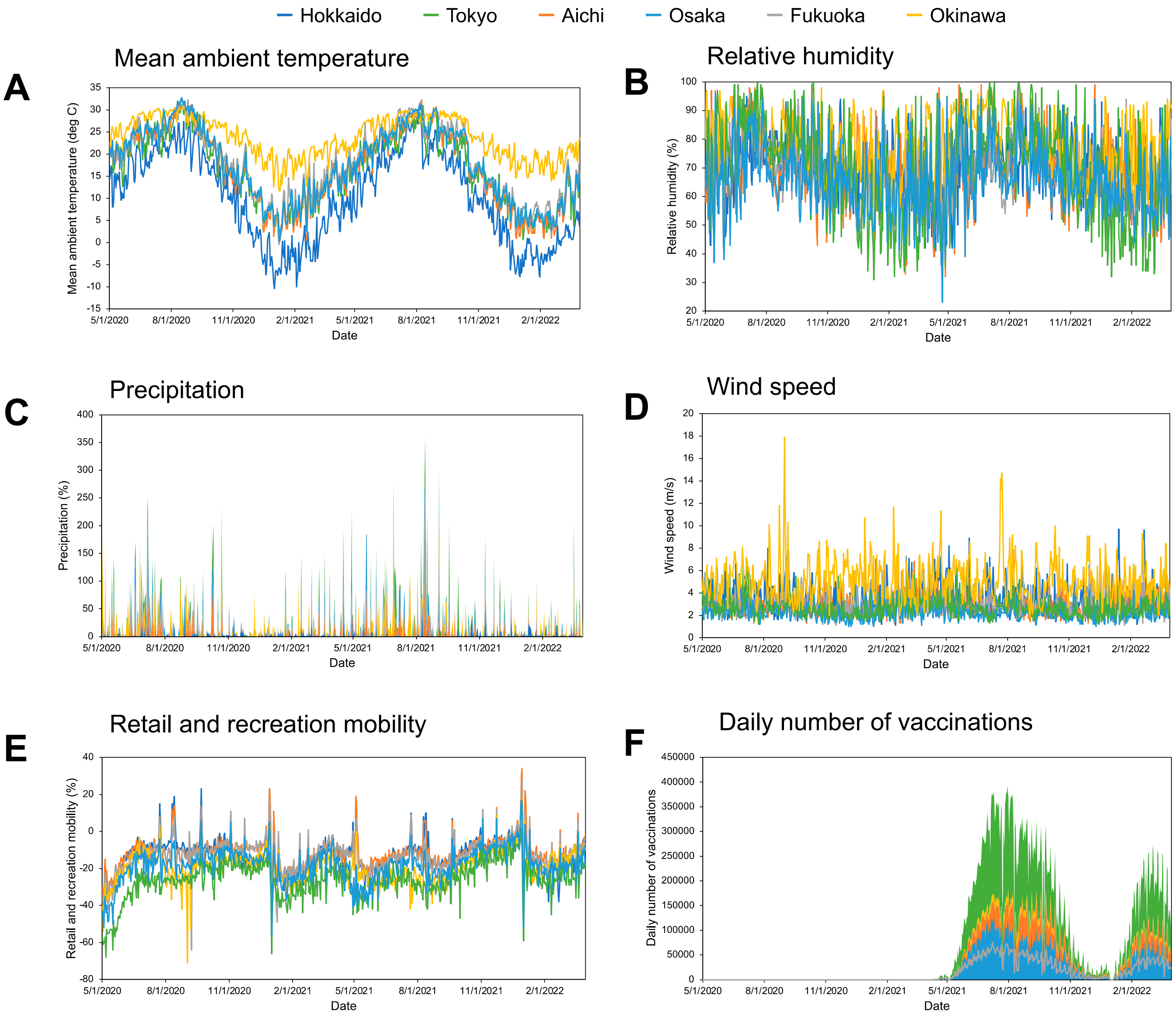

Figure S3. Time-series plots of daily meteorological variables and mobility patterns across all six selected prefectures over the study duration. (A) Time-series plot of daily mean ambient temperature in Hokkaido, Tokyo, Aichi, Osaka, Fukuoka, and Okinawa, Japan (units: °C); (B) Time-series plot of daily relative humidity in Hokkaido, Tokyo, Aichi, Osaka, Fukuoka, and Okinawa, Japan (units: %); (C) Time-series plot of daily precipitation in Hokkaido, Tokyo, Aichi, Osaka, Fukuoka, and Okinawa, Japan (units: mm); (D) Time-series plot of daily wind speed in Hokkaido, Tokyo, Aichi, Osaka, Fukuoka, and Okinawa, Japan (units: m/s); (E) Time-series plot of daily retail and recreation mobility across all prefectures and days (units: %); and (F) Time-series plot of daily number of vaccinations in Hokkaido, Tokyo, Aichi, Osaka, Fukuoka, and Okinawa, Japan (units: doses). Notes: the present study covers the period between 1 May 2020 and 31 March 2022 and included six prefectures (i.e., Hokkaido, Tokyo, Aichi, Osaka, Fukuoka, and Okinawa). Hokkaido, Tokyo, Aichi, Osaka, Fukuoka, and Okinawa are represented in dark blue, green, light red, light blue, grey, and yellow, respectively.

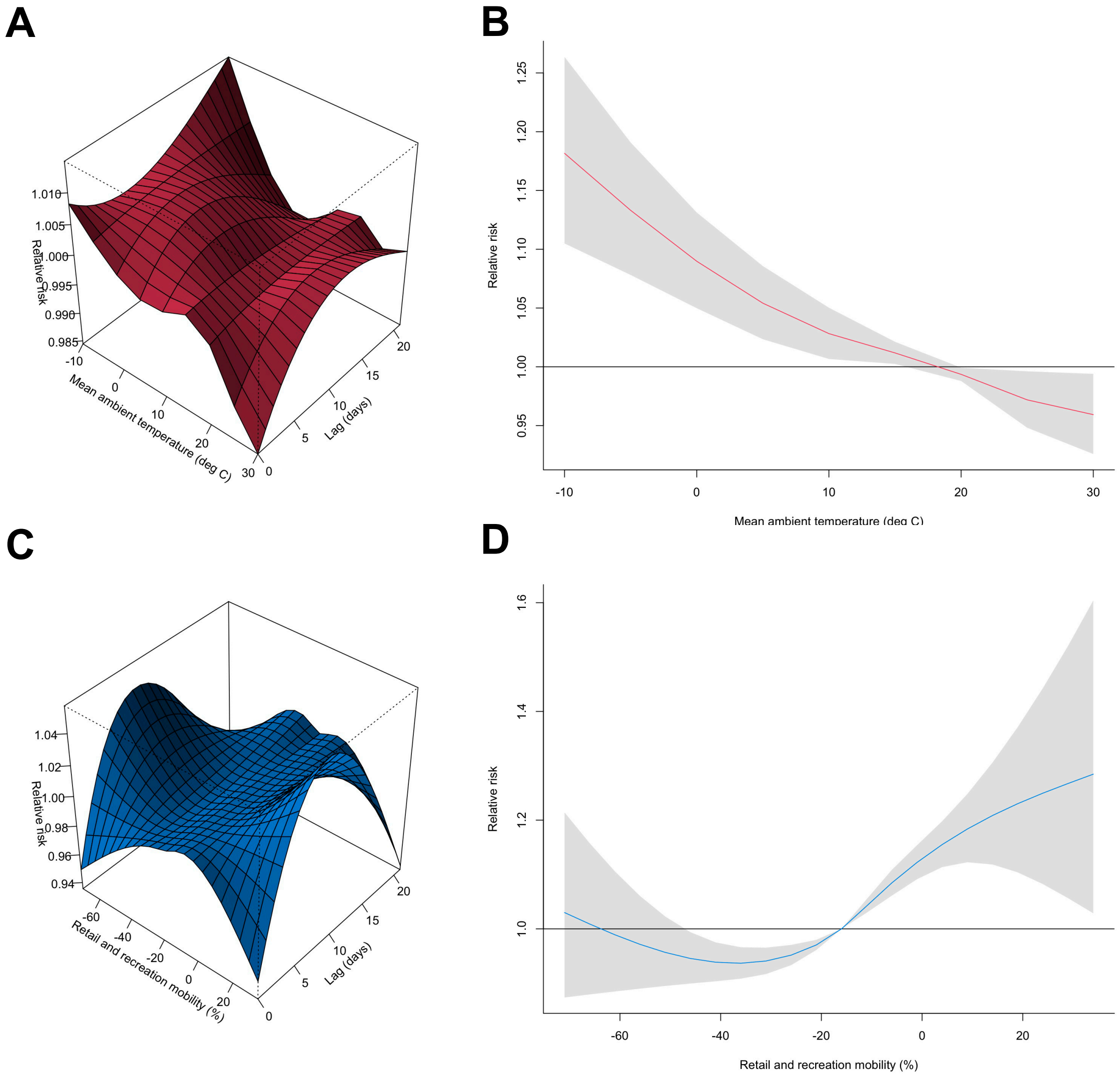

Figure S4. Visualizing pooled non-linear and delayed associations of the relative risks of the time-dependent transmissibility of SARS-CoV-2 with mean ambient temperature and retail and recreation mobility. (A) Three-dimensional plot of the association between daily mean ambient temperature (units: °C) and the percent change in the estimated time-dependent effective reproductive number (R_t) over a lag of 21 days. (B) Overall associations of the 21-day cumulative risk of the percent change in the estimated time-dependent R_t of SARS-CoV-2 with daily mean ambient temperature (units: °C). (C) Three-dimensional plot of the association between daily retail and recreation mobility (units: %) and the percent change in the estimated time-dependent R_t over a period of 21 days. (D) Overall associations of the 21-day cumulative risk of the percent change in the estimated time-dependent R_t of SARS-CoV-2 with daily retail and recreation mobility (units: %). Red and blue lines represent the estimated cumulative relative risks of the time-dependent transmissibility of SARS-CoV-2, with shaded bands as 95% CIs. The corresponding reference levels were 18.5 °C and −16.0%, respectively. The present study covers the period between 1 May 2020 and 31 March 2022 in six selected prefectures (i.e., Hokkaido, Tokyo, Aichi, Osaka, Fukuoka, and Okinawa) in Japan. In this sensitivity analysis, a natural cubic spline of time was set up with different degrees of freedom (three degrees of freedom per year).

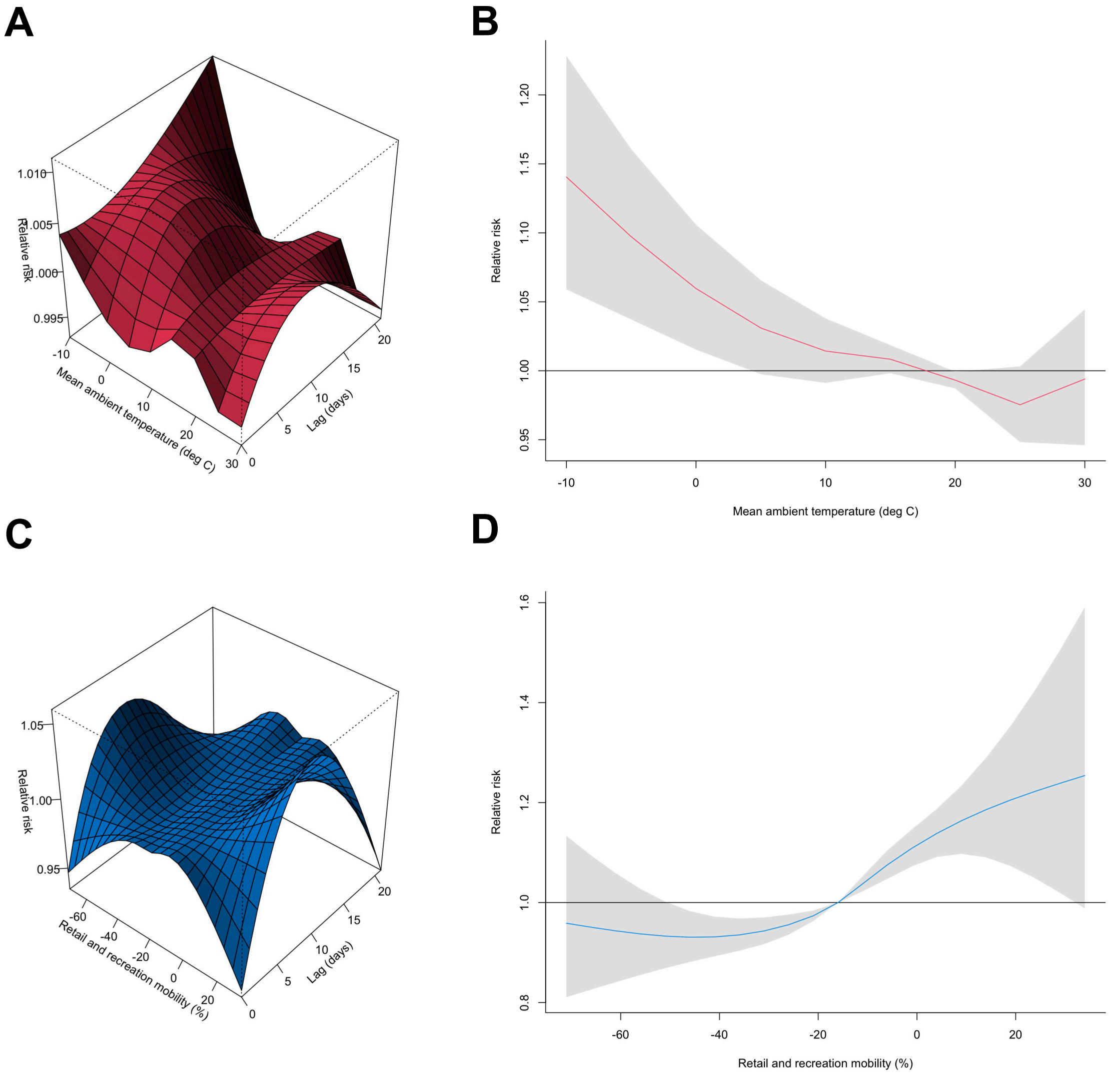

Figure S5. Visualizing pooled non-linear and delayed associations of the relative risks of the time-dependent SARS-CoV-2 transmissibility with mean ambient temperature and retail and recreation mobility. (A) Three-dimensional plot of the association between daily mean ambient temperature (units: °C) and the percent change in estimated time-dependent effective reproductive number (R_t) over a lag of 21 days. (B) Overall associations of the 21-day cumulative risk of the percent change in estimated time-dependent R_t of SARS-CoV-2 with daily mean ambient temperature (units: °C). (C) Three-dimensional plot of the association between daily retail and recreation mobility (units: %) and the percent change in estimated time-dependent R_t over a lag of 21 days. (D) Overall associations of the 21-day cumulative risk of the percent change in the estimated time-dependent R_t of SARS-CoV-2 with daily retail and recreation mobility (units: %). Red and blue lines represent the estimated cumulative relative risks of the time-dependent transmissibility of SARS-CoV-2, with shaded bands as 95% CIs. The corresponding reference levels were 18.5 °C and −16.0%, respectively. The present study covers the period between 1 May 2020 and 31 March 2022 in six selected prefectures (i.e., Hokkaido, Tokyo, Aichi, Osaka, Fukuoka, and Okinawa) in Japan. In this sensitivity analysis, a natural cubic spline of time was set up with different degrees of freedom (eleven degrees of freedom per year).

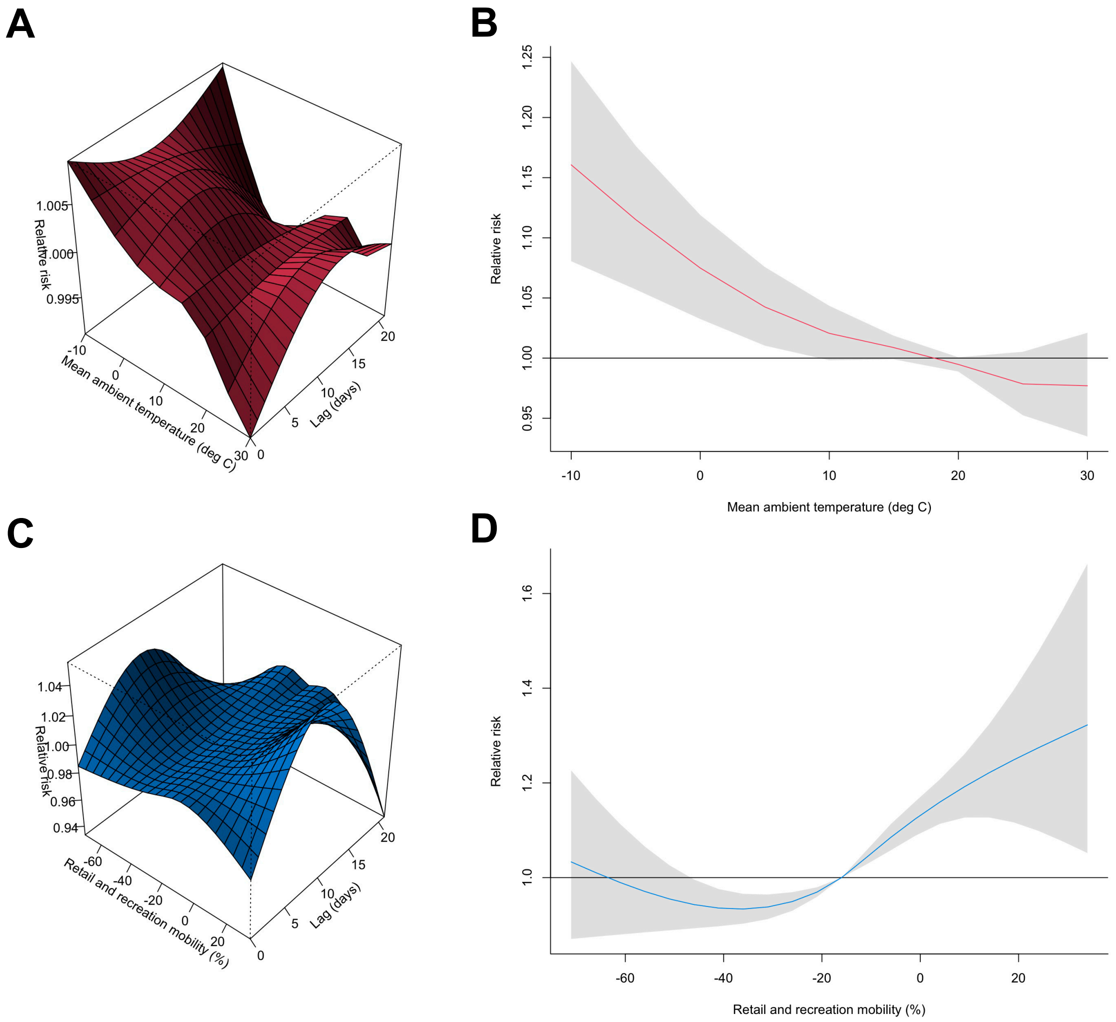

Figure S6. Visualizing pooled non-linear and delayed associations of the relative risks of time-dependent SARS-CoV-2 transmissibility with mean ambient temperature and retail and recreation mobility. (A) Three-dimensional plot of the association between daily mean ambient temperature (units: °C) and the percent change in estimated time-varying effective reproductive number (R_t) over a lag of 21 days. (B) Overall associations of the 21-day cumulative risk of the percent change in estimated time-varying R_t of SARS-CoV-2 with daily mean ambient temperature (units: °C). (C) Three-dimensional plot of the association between daily retail and recreation mobility (units: %) and the percent change in estimated time-dependent R_t over a lag of 21 days. (D) Overall associations of the 21-day cumulative risk of the percent change in the estimated time-dependent R_t of SARS-CoV-2 with daily retail and recreation mobility (units: %). Red and blue lines represent the estimated cumulative relative risks of the time-dependent SARS-CoV-2 transmissibility, with shaded bands as 95% CIs. The corresponding reference levels were 18.5 °C and −16.0%, respectively. The present study covers the period between 1 May 2020 and 31 March 2022 in six selected prefectures (i.e., Hokkaido, Tokyo, Aichi, Osaka, Fukuoka, and Okinawa) in Japan. In this sensitivity analysis, the lag-response dimension using a natural cubic spline and three equally placed internal knots was redefined.

Table S1. Descriptive statistics for daily new confirmed COVID-19 cases, effective reproductive numbers, meteorological variables, mobility patterns, and vaccinations across all days by prefecture.

Prefecture	Potential drivers	Mean	SD	Min	P_{25}	P_{50}	P_{75}	Max
Hokkaido	Daily new confirmed cases	302	302	0	13	63	201	4,295
	Effective reproductive number	1.16	0.69	0.18	0.76	1.00	1.43	5.54
	Mean ambient temperature (°C)	10.22	9.94	–10.4	–7.55	10.4	19.2	29.2
	Relative humidity (%)	70.90	10.07	36 .00	64.50	72.00	78.00	95.00
	Precipitation (mm)	2.80	6.66	0.00	0.00	0.00	2.50	61.5
	Wind speed (m/s)	3.43	1.41	1.1	2.40	3.20	4.30	9.70
	Retail and recreation mobility (%)	–12.64	9.00	–53.00	–18.00	–12.00	–8.00	23.00
	Daily number of vaccinations	14,410	20,546	0	0	31	28,861	73,295
Tokyo	Daily new confirmed cases	1,753	3,694	2	124	352	966	20,040
	Effective reproductive number	1.17	0.86	0.23	0.81	0.81	1.29	9.15
	Mean ambient temperature (°C)	16.57	7.89	0.70	9.70	16.70	22.90	31.70
	Relative humidity (%)	69.58	16.91	31.00	56.00	72.00	82.00	100.00
	Precipitation (mm)	4.67	13.77	0.00	0.00	0.00	1.75	138.50
	Wind speed (m/s)	2.71	.84	1.2	2.10	2.60	3.10	6.5
	Retail and recreation mobility (%)	–26.21	9.88	–68.00	–30.50	–26.00	–20.00	2.00
	Daily number of vaccinations	38,887	58,195	0	0	446	74,600	228,078
Aichi	Daily new confirmed cases	948	2,562	0	21	86	290	16,199
	Effective reproductive number	1.40	1.81	0.11	0.67	0.95	1.45	16.35
	Mean ambient temperature (°C)	16.91	8.59	0.00	9.50	16.90	24.45	32.50
	Relative humidity (%)	69.18	13.04	24.00	61.00	68.50	76.00	99.00
	Precipitation (mm)	4.97	12.71	0.00	0.00	0.00	1.75	101.50
	Wind speed (m/s)	2.96	1.04	1.20	2.20	2.70	3.60	7.90
	Retail and recreation mobility (%)	–11.2	8.27	–41.00	–16.00	–11.00	–7.00	34.00
	Daily number of vaccinations	20,295	30,065	0	0	14	40,019	114,952
Osaka	Daily new confirmed cases	721	1,365	0	55	166	618	7,872
	Effective reproductive number	1.21	0.87	0.27	0.69	0.93	1.46	6.75
	Mean ambient temperature (°C)	17.59	8.26	0.20	10.30	17.60	24.90	32.80
	Relative humidity (%)	65.56	11.49	23.00	58.00	65.00	72.00	96.00
	Precipitation (mm)	4.76	12.82	0.00	0.00	0.00	1.00	105.00
	Wind speed (m/s)	2.32	0.74	1.00	1.80	2.20	2.70	5.20
	Retail and recreation mobility (%)	–18.02	9.14	–56.00	–23.00	–17.00	–12.50	17.00
	Daily number of vaccinations	23,259	33,884	0	0	112	42,947	125,443
Fukuoka	Daily new confirmed cases	358	789	0	9	46	219	5,122
	Effective reproductive number	1.32	1.37	0.11	0.64	0.93	1.54	12.66
	Mean ambient temperature (°C)	18.00	7.94	–0.50	11.00	18.30	24.75	32.30
	Relative humidity (%)	68.47	10.96	42.00	61.00	68.00	76.00	98.00
	Precipitation (mm)	5.58	18.62	0.00	0.00	0.00	1.00	231.50
	Wind speed (m/s)	2.83	1.03	1.10	2.10	2.60	3.30	7.80
	Retail and recreation mobility (%)	–12.67	8.82	–64.00	–17.00	–12.00	–8.00	28.00
	Daily number of vaccinations	13,965	20,361	0	0	23	26,965	74,951
Okinawa	Daily new confirmed cases	174	308	0	13	39	140	1,819
	Effective reproductive number	1.54	2.12	0.04	0.71	1.04	1.48	20.63
	Mean ambient temperature (°C)	23.79	4.59	11.60	20.20	24.10	28.10	30.50
	Relative humidity (%)	77.91	10.59	52.00	70.00	80.00	86.00	98.00
	Precipitation (mm)	7.33	20.72	0.00	0.00	0.00	3.50	213.00
	Wind speed (m/s)	5.16	1.85	1.80	3.90	4.90	6.10	17.90
	Retail and recreation mobility (%)	–19.21	8.65	–71.00	–25.00	–19.00	–14.00	18.00
	Daily number of vaccinations	3,449	5,277	0	0	32	5,735	23,275

Abbreviations: SD, standard deviation; Min, minimum; P_{25}, 25th percentile; P_{25}, 50th percentile; P_{75}, 75th percentile; Max, maximum. Notes: The present study covers the period between May 1, 2020, and March 31, 2022, in six selected prefectures (i.e., Hokkaido, Tokyo, Aichi, Osaka, Aichi, Fukuoka, and Okinawa) in Japan.

Table S2. Assessments of multi-collinearity using pairwise Spearman's rank-order linear correlation matrix between daily meteorological variables, mobility patterns, and vaccinations across all six prefectures and the entire study period.

Potential drivers	1	2	3	4	5	6	7
1. Effective reproductive number	1.00						
2. Mean ambient temperature (°C)	−0.04**	1.00					
3. Relative humidity (%)	0.003	0.38***	1.00				
4. Precipitation (mm)	0.01	0.06***	0.62***	1.00			
5. Wind speed (m/s)	0.01	0.13***	0.06***	0.11***	1.00		
6. Retail and recreation mobility (%)	0.13***	−0.12***	−0.20***	−0.19***	−0.06***	1.00	
7. Daily number of vaccinations	−0.10***	0.07***	0.02	0.01	−0.10***	−0.04**	1.00

Notes: The present study covers the period between May 1, 2020, and March 31, 2022, in six selected prefectures (i.e., Hokkaido, Tokyo, Aichi, Osaka, Aichi, Fukuoka, and Okinawa) in Japan. Significant predictors in the statistical model are described by *P < 0.05, **P < 0.01, and ***P < 0.001.

References

1. Cori, A.; Ferguson, N. M.; Fraser, C.; Cauchemez, S., A new framework and software to estimate time-varying reproduction numbers during epidemics. *Am. J. Epidemiol.* **2013,** 178, (9), 1505–12.
2. Fraser, C., Estimating individual and household reproduction numbers in an emerging epidemic. *PLoS One* **2007,** 2, (8), e758.
3. Cowling, B.J.; Ali, S.T.; Ng, T.W.Y.; Tsang, T.K.; Li, J.C.M.; Fong, M.W.; Liao, Q.; Kwan, M.Y.; Lee, S.L.; Chiu, S.S.; et al. Impact assessment of non-pharmaceutical interventions against coronavirus disease 2019 and influenza in Hong Kong: an observational study. *Lancet Public Health* **2020**, *5*, e279–e288, doi:10.1016/s2468-2667(20)30090-6.
4. Ali, S. T.; Cowling, B. J.; Wong, J. Y.; Chen, D.; Shan, S.; Lau, E. H. Y.; He, D.; Tian, L.; Li, Z.; Wu, P., Influenza seasonality and its environmental driving factors in mainland China and Hong Kong. *Sci. Total Environ.* **2022,** 818, 151724.
5. Ali, S. T.; Kadi, A. S.; Ferguson, N. M., Transmission dynamics of the 2009 influenza A (H1N1) pandemic in India: the impact of holiday-related school closure. *Epidemics* **2013,** 5, (4), 157–163.
6. Wallinga, J.; Teunis, P., Different epidemic curves for severe acute respiratory syndrome reveal similar impacts of control measures. *Am. J. Epidemiol.* **2004,** 160, (6), 509–16.
7. Nishiura, H.; Linton, N. M.; Akhmetzhanov, A. R., Serial interval of novel coronavirus (COVID-19) infections. *Int. J. Infect. Dis.* **2020,** 93, 284–286.
8. Chan, Y. H. 2003 Biostatistics 104: correlational analysis. *Singapore Med. J.* **2003**, *44*, 614–619.
9. Lin, H.; Tao, J.; Kan, H.; Qian, Z.; Chen, A.; Du, Y.; Liu, T.; Zhang, Y.; Qi, Y.; Ye, J.; Li, S.; Li, W.; Xiao, J.; Zeng, W.; Li, X.; Stamatakis, K. A.; Chen, X.; Ma, W., Ambient particulate matter air pollution associated with acute respiratory distress syndrome in Guangzhou, China. *J. Expo. Sci. Environ. Epidemiol.* **2018,** 28, (4), 392–399.
10. Liu, K.; Hou, X.; Ren, Z.; Lowe, R.; Wang, Y.; Li, R.; Liu, X.; Sun, J.; Lu, L.; Song, X.; Wu, H.; Wang, J.; Yao, W.; Zhang, C.; Sang, S.; Gao, Y.; Li, J.; Li, J.; Xu, L.; Liu, Q., Climate factors and the East Asian summer monsoon may drive large outbreaks of dengue in China. *Environ. Res.* **2020,** 183, 109190.
11. Wu, X.; Lang, L.; Ma, W.; Song, T.; Kang, M.; He, J.; Zhang, Y.; Lu, L.; Lin, H.; Ling, L., Non-linear effects of mean temperature and relative humidity on dengue incidence in Guangzhou, China. *Sci. Total Environ.* **2018,** 628–629, 766–771.
12. Talmoudi, K.; Bellali, H.; Ben-Alaya, N.; Saez, M.; Malouche, D.; Chahed, M. K., Modeling zoonotic cutaneous leishmaniasis incidence in central Tunisia from 2009-2015: Forecasting models using climate variables as predictors. *PLoS Negl. Trop. Dis.* **2017,** 11, (8), e0005844.
13. Xie, J.; Zhu, Y., Association between ambient temperature and COVID-19 infection in 122 cities from China. *Sci. Total Environ.* **2020,** 724, 138201.
14. Gasparrini, A.; Masselot, P.; Scortichini, M.; Schneider, R.; Mistry, M. N.; Sera, F.; Macintyre, H. L.; Phalkey, R.; Vicedo-Cabrera, A. M., Small-area assessment of temperature-related mortality risks in England and Wales: a case time series analysis. *Lancet Planet. Health* **2022,** 6, (7), e557–e564.
15. Gasparrini, A.; Guo, Y.; Hashizume, M.; Lavigne, E.; Zanobetti, A.; Schwartz, J.; Tobias, A.; Tong, S.; Rocklöv, J.; Forsberg, B.; Leone, M.; De Sario, M.; Bell, M. L.; Guo, Y. L.; Wu, C. F.; Kan, H.; Yi, S. M.; de Sousa Zanotti Stagliorio Coelho, M.; Saldiva, P. H.; Honda, Y.; Kim, H.; Armstrong, B., Mortality risk attributable to high and low ambient temperature: a multicountry observational study. *Lancet* **2015,** 386, (9991), 369–375.
16. Gasparrini, A; Armstrong, B; Kenward, MG. Distributed lag non-linear models. *Stat. Med.* **2010**, *29*, 2224–2234.
17. Guan, W. J.; Ni, Z. Y.; Hu, Y.; Liang, W. H.; Ou, C. Q.; He, J. X.; Liu, L.; Shan, H.; Lei, C. L.; Hui, D. S. C.; Du, B.; Li, L. J.; Zeng, G.; Yuen, K. Y.; Chen, R. C.; Tang, C. L.; Wang, T.; Chen, P. Y.; Xiang, J.; Li, S. Y.; Wang, J. L.; Liang, Z. J.; Peng, Y. X.; Wei, L.; Liu, Y.; Hu, Y. H.; Peng, P.; Wang, J. M.; Liu, J. Y.; Chen, Z.; Li, G.; Zheng, Z. J.; Qiu, S. Q.; Luo, J.; Ye, C. J.; Zhu,

S. Y.; Zhong, N. S., Clinical Characteristics of Coronavirus Disease 2019 in China. *N. Engl. J. Med.* **2020,** 382, (18), 1708–1720.
18. Li, Q.; Guan, X.; Wu, P.; Wang, X.; Zhou, L.; Tong, Y.; Ren, R.; Leung, K. S. M.; Lau, E. H. Y.; Wong, J. Y.; Xing, X.; Xiang, N.; Wu, Y.; Li, C.; Chen, Q.; Li, D.; Liu, T.; Zhao, J.; Liu, M.; Tu, W.; Chen, C.; Jin, L.; Yang, R.; Wang, Q.; Zhou, S.; Wang, R.; Liu, H.; Luo, Y.; Liu, Y.; Shao, G.; Li, H.; Tao, Z.; Yang, Y.; Deng, Z.; Liu, B.; Ma, Z.; Zhang, Y.; Shi, G.; Lam, T. T. Y.; Wu, J. T.; Gao, G. F.; Cowling, B. J.; Yang, B.; Leung, G. M.; Feng, Z., Early Transmission Dynamics in Wuhan, China, of Novel Coronavirus-Infected Pneumonia. *N. Engl. J. Med.* **2020,** 382, (13), 1199–1207.
19. Lauer, S. A.; Grantz, K. H.; Bi, Q.; Jones, F. K.; Zheng, Q.; Meredith, H. R.; Azman, A. S.; Reich, N. G.; Lessler, J., The Incubation Period of Coronavirus Disease 2019 (COVID-19) From Publicly Reported Confirmed Cases: Estimation and Application. *Ann. Intern. Med.* **2020,** 172, (9), 577–582.
20. Linton, N. M.; Kobayashi, T.; Yang, Y.; Hayashi, K.; Akhmetzhanov, A. R.; Jung, S. M.; Yuan, B.; Kinoshita, R.; Nishiura, H., Incubation Period and Other Epidemiological Characteristics of 2019 Novel Coronavirus Infections with Right Truncation: A Statistical Analysis of Publicly Available Case Data. *J. Clin. Med.* **2020,** 9, (2), 538.
21. Azuma, K.; Kagi, N.; Kim, H.; Hayashi, M., Impact of climate and ambient air pollution on the epidemic growth during COVID-19 outbreak in Japan. *Environ. Res.* **2020,** 190, 110042.
22. Hayashi, K.; Kayano, T.; Anzai, A.; Fujimoto, M.; Linton, N.; Sasanami, M.; Suzuki, A.; Kobayashi, T.; Otani, K.; Yamauchi, M.; Suzuki, M.; Nishiura, H., Assessing Public Health and Social Measures Against COVID-19 in Japan From March to June 2021. *Front. Med.* **2022,** 9, 937732.
23. Jones, P. J.; Koolhof, I. S.; Wheeler, A. J.; Williamson, G. J.; Lucani, C.; Campbell, S. L.; Bowman, D.; Cooling, N.; Gasparrini, A.; Johnston, F. H., Characterising non-linear associations between airborne pollen counts and respiratory symptoms from the AirRater smartphone app in Tasmania, Australia: A case time series approach. *Environ. Res.* **2021,** 200, 111484.
24. Amuakwa-Mensah, F.; Marbuah, G.; Mubanga, M., Climate variability and infectious diseases nexus: Evidence from Sweden. *Infect. Dis. Model.* **2017,** 2, (2), 203–217.
25. Zhu, Y.; Xie, J.; Huang, F.; Cao, L., The mediating effect of air quality on the association between human mobility and COVID-19 infection in China. *Environ. Res.* **2020,** 189, 109911.
26. Shao, W.; Xie, J.; Zhu, Y., Mediation by human mobility of the association between temperature and COVID-19 transmission rate. *Environ. Res.* **2021,** 194, 110608.
27. Imai, C.; Armstrong, B.; Chalabi, Z.; Mangtani, P.; Hashizume, M., Time series regression model for infectious disease and weather. *Environ. Res.* **2015,** 142, 319-27.
28. Jung, S.M.; Endo, A.; Akhmetzhanov, A.R.; Nishiura, H. Predicting the effective reproduction number of COVID-19: inference using human mobility, temperature, and risk awareness. *Int. J. Infect. Dis.* **2021**, *113*, 47–54, doi:10.1016/j.ijid.2021.10.007.
29. Schneider, C; Dryhurst, S; Kerr, J; Freeman, A; Recchia, G; Spiegelhalter, D; Linden, S. COVID-19 risk perception: a longitudinal analysis of its predictors and associations with health protective behaviours in the United Kingdom. *J. Risk Res.* **2021**, *24*, 294–313.
30. Cabinet Relations Office. Advisory committee for COVID-19. 2020. Available online: https://www.cas.go.jp/jp/seisaku/ful/bunkakai/kongo_soutei_taisaku.pdf (accessed on 1 July 2022).
31. Prefecture/city/town/village. Population data. Available online: https://uub.jp/rnk/rnk.cgi?T=p&S=j&B=20201001 (Accessed on 1 July 2022).
32. Gasparrini, A., Distributed Lag Linear and Non-Linear Models in R: The Package dlnm. *J. Stat. Softw.* **2011,** 43, (8), 1–20.
33. Wood, S.N. Generalized Additive Models: An Introduction with R (2nd Edition). *Chapman & Hall/CRC* **2017**, doi:10.1201/9781315370279
